# Virus-like vaccines against HIV/SIV synergize with a subdominant antigen T cell vaccine

**DOI:** 10.1186/s12967-019-1924-1

**Published:** 2019-05-24

**Authors:** Melanie Schwerdtfeger, Anne-Marie Carola Andersson, Lasse Neukirch, Peter Johannes Holst

**Affiliations:** 1 Centre for Medical Parasitology, Department of Immunology and Microbiology, University of Copenhagen, Mærsk Tower 07-11, Blegdamsvej 3B, 2200 Copenhagen N, Denmark; 2 Present Address: Department of Experimental Medicine, University of Campania “Luigi Vanvitelli”, Via L. Armanni 5, 80138 Naples, Italy; 3 InProTherApS, BioInnovation Institute, COBIS, Ole Maaløes Vej 3, 2200 Copenhagen N, Denmark; 4 Present Address: Clinical Cooperation Unit “Applied Tumor Immunity”, National Center for Tumor Diseases (NCT) and German Cancer Research Center (DKFZ), Im Neuenheimer Feld 460, 69120 Heidelberg, Germany

**Keywords:** Adenoviral vectors, Human immunodeficiency virus, Virus-like particles, Virus-like vaccines, T-cells, Antibodies, Heterologous viral vectored prime-boost immunization, Subdominant antigen vaccine

## Abstract

**Background:**

In non-human primates (NHPs) and humans, partial protection from HIV/SIV infection or suppression of replication is achievable by Env-binding antibodies and Gag-specific CD8+ T-cells targeting protective epitopes. Unfortunately, such T-cell responses are frequently dominated by responses to non-protective, variable epitopes. In this study we attempt to combine three independent approaches, each developed to prevent immunodominance of non-protective epitopes. These approaches were (1) vaccines consisting exclusively of putatively protective p24 Gag highly conserved elements (CEs), (2) vaccines using solely subdominant antigens which were acutely protective in a recent NHP trial, and (3) virus-encoded virus-like particle vaccines (virus-like vaccines/VLVs) using heterologous Env and Gag sequences to enable selection of broadly cross-reactive responses and to avoid immunodominance of non-conserved sequences in prime-boost regimens as previously observed.

**Methods:**

We vaccinated outbred CD1 mice with HIV-1 clade B Gag/Env encoded in an adenoviral prime and SIVmac239 Gag/Env in an MVA boost. We combined this completely heterologous immunization regimen and the homologous SIVmac239 Gag/Env immunization regimen with an additional prime encoding SIV CEs and accessory antigens Rev, Vif and Vpr (Ad-Ii-SIVCErvv). T-cell responses were analyzed by intracellular cytokine staining of splenocytes and antibody responses by trimer-specific ELISA, avidity and isotype-specific ELISA.

**Results:**

Env dominance could be avoided successfully in the completely heterologous prime-boost regimen, but Env immunodominance reappeared when Ad-Ii-SIVCErvv was added to the prime. This regimen did however still induce more cross-reactive Gag-specific CD8+ T-cells and Env-specific antibodies. Including Ad-Ii-SIVCErvv in the homologous prime-boost not only elicited accessory antigen-specific CD8+ memory T-cells, but also significantly increased the ratio of Gag- to Env-specific CD8+ T-cells. The CD4+ T-cell response shifted away from structural antigens previously associated with infection-enhancement.

**Conclusion:**

The homologous Gag/Env prime-boost with Ad-Ii-SIVCErvv prime combined acutely protective CD8+ T-cell responses to subdominant antigens and Env-binding antibodies with chronically protective Gag-specific CD8+ T-cells in outbred mice. This vaccine regimen should be tested in an NHP efficacy trial.

**Electronic supplementary material:**

The online version of this article (10.1186/s12967-019-1924-1) contains supplementary material, which is available to authorized users.

## Background

The primary goal of a human immunodeficiency virus type 1 (HIV-1) vaccine is to protect from HIV-1 acquisition [1]. The partial protection in the HIV vaccination trial RV144 correlated with IgG antibodies targeting the V1V2 regions of the viral envelope (Env) protein [2, 3]. If prevention of infection is not possible an alternative goal is to induce CD8+ T-cells that can control the infection [1]. In chronically infected individuals CD8+ T-cells targeting group-specific antigen (Gag) correlated directly with a lower viral load while Env-specific CD8+ T-cells correlated inversely [4, 5]. These associations provide an incentive to increase antibody responses towards Env as well as CD8+ T-cell responses towards Gag and to reduce Env-specific CD8+ T-cell responses.

A great impediment for the development of an HIV-1 vaccine that induces protective immune responses or such that can control HIV-1 infection is the immunodominance of non-protective, variable epitopes which the virus can easily mutate without fitness cost. An immune response towards these epitopes suppresses responses towards more conserved regions with a higher protective potential [6]. Kulkarni et al. addressed this problem by designing immunogens based on 7 highly conserved elements (CEs) in HIV-1 p24 Gag [7]. A DNA vaccine encoding this string of peptides raised higher T-cell responses to the CEs compared to vaccination with full-length Gag DNA in mice and macaques [7, 8]. A prime vaccination using CE DNA, followed by a boost with full-length Gag DNA, increased the magnitude and breadth of Gag-specific T-cell responses, including responses to the CEs [8]. In Hu et al. simian immunodeficiency virus (SIV) p27 Gag CEs were described in the same positions as the HIV-1 p24 CEs [9]. The SIV p27 Gag CE antigen in combination with an HIV-1 Env CE DNA vaccine in macaques resulted in potent CE-specific CD8+ T-cell responses and did not show control of viremia but a reverse correlation of p27 Gag CE-specific CD8+ T-cells and peak viremia [10].

We approached the objective of focusing the T-cell response on conserved parts of Gag from a different angle in Andersson and Holst [11]. We vaccinated outbred mice with virus-like vaccines (VLVs), i.e. virus-vectored virus-like particle (VLP) vaccines, encoding Gag and Env. Human adenovirus type 5 (Ad) served as a prime and modified vaccinia virus Ankara (MVA) as a boost vector. The origin of Gag was varied between HIV-1 consensus clade B (HIVconB) in the prime and SIVmac239 in the boost to selectively expand CD8+ T-cell responses to epitopes shared between the heterologous Gags. For comparison, a second group received homologous SIVmac239 Gag in both prime and boost immunization. All VLVs encoded SIVmac239 Env downstream of Gag to induce Env-specific antibody responses which have been shown to benefit from Env-presentation on VLPs [12–14]. In mice vaccinated with homologous Gag higher and broader Gag-specific CD8+ T-cell responses and more Env-specific antibodies were detected compared to mice receiving heterologous Gag. Furthermore, the mice in the homologous Gag group raised more Gag- than Env-specific CD8+ T-cells. The opposite was true for differing Gags, which suggests immunodominance of the homologous Env over the heterologous Gag [11].

The first antigens targeted by CD8+ T-cells in a natural HIV-1 infection are often Env and Nef followed by p24 Gag and DNA polymerase (Pol). To completely avoid these immunodominant antigens during vaccination, we chose the approach described in Xu et al. [15]: only the SIVmac239 subdominant accessory antigens trans-activator of transcription (Tat), viral infectivity factor (Vif), regulator of expression of virion proteins (Rev) and viral protein R (Vpr) were encoded in adenovirus-vectored vaccines. To increase the intrinsically low immunogenicity of these antigens, we included the genetic T-cell adjuvant MHC class II-associated invariant chain (Ii). An invariant chain vaccine has been shown to induce a degree of acute protection in a rodent LCMV model which enabled the infection to broaden the response to dominant antigens [16]. In combination with the SIVmac239 subdominant accessory antigens, the vaccination regimen induced accessory antigen-specific CD8+ T-cells in a prime and boost immunization of non-human primates (NHPs) which were subsequently challenged by SIV. The vaccinated NHPs showed profound control of acute infection (2/6 completely controlling infection following 10 challenges) and all vaccinated animals had long-term immunological benefits such as reduced rectal CD4+ T-cell depletion and highly limited CD8+ T-cell hyperactivation [15].

Also in Martins et al. rhesus macaques vaccinated with the accessory antigens Vif, Rev, Tat and negative factor (Nef) became detectably infected at a slower rate than controls and animals that received accessory antigens in combination with Gag and/or Env [17]. Interestingly, immunization with all these antigens led to the best control of chronic viremia after SIV acquisition. In Hel et al. Rev, Tat and Nef were added to a vaccine consisting of Gag, Pol and Env. This resulted in a delay and reduction of SIV viremia in NHPs, a better preservation of virus-specific CD4+ T-cells and increased survival [18]. These findings suggest that combining the immunodominant antigens Gag and Env with subdominant, accessory antigens could result in better protection and viral control, with the *caveat* that Env-specific T-cell responses are not protection-associated and are immunodominant.

In this study we aimed to overcome the Env immunodominance over Gag observed in Andersson and Holst by using both heterologous Gag and Env in a VLV Ad-prime MVA-boost regimen in outbred mice. A positive side effect of additionally varying Env between prime and boost could be the induction of broader antibody responses for protection against infection. We also hypothesized that including CEs could help to focus the T-cell response on more protective Gag epitopes, ultimately leading to better long-term control of viremia. In addition, we assessed if a combination of the immunodominant Gag and Env antigens with subdominant accessory antigens could result in responses associated with protection as suggested by Xu et al., Martins et al. and Hel et al. [15, 17, 18]. To this end, we combined the homologous Gag/Env immunization from Andersson and Holst and the heterologous Gag/Env vaccination with an additional Ad prime encoding Ii, SIV p27 Gag CEs and SIVmac239 Rev, Vif and Vpr (Ad-Ii-SIVCErvv).

In the heterologous Gag/Env prime-boost regimen Env dominance could be successfully avoided. When including Ad-Ii-SIVCErvv Env dominance reappeared, but nevertheless more cross-reactive Gag-specific CD8+ T-cell and antibody responses to Env were elicited. Adding Ad-Ii-SIVCErvv to the homologous Gag/Env prime-boost not only induced accessory antigen-specific CD8+ T-cell memory responses, but also significantly increased the ratio of Gag- to Env-specific CD8+ T-cells. The CD4+ T-cell response further shifted away from the structural antigens previously associated with infection-enhancement [19]. We can conclude that adding Ad-Ii-SIVCErvv to the homologous Gag/Env prime-boost regimens acted synergistically to induce diverse responses, each which have previously been associated with partial protection independently.

## Methods

### Mice

Female CD1 mice were obtained from Envigo (United Kingdom) at the age of 6–8 weeks and allowed to acclimatize for 1 week before an experiment was initiated. All experiments were performed according to national guidelines and experimental protocols approved by the national animal experiments inspectorate (Dyreforsøgstilsynet, permit ID: 2016-15-0201-01131).

### Viral vector production

The expression cassettes for adenovirus production consisted of the genes of interest under the control of a CMV promoter, a Tet operator and a simian virus 40 polyadenylation signal. For the 2 adenoviral virus-like vaccines, HIV-1 consensus clade B Gag and HIV-1 JR-FL Env (resulting virus: Ad-HIVB) or SIVmac239 Gag and Env (resulting virus: Ad-SIV, used in Andersson and Holst [11]) were linked by a glycine-serine-glycine linker and a self-cleavable porcine teschovirus-1 2A (P2A) peptide. For the additional adenoviral vector, the C-terminus of the mouse Ii (aa 1–75) was fused to SIV p27 CEs and Rev, Vif and Vpr from SIVmac239 (resulting virus: Ad-Ii-SIVCErvv). These were encoded in the expression cassette, which was inserted into the E1 region of an E1/E3-deleted replication-deficient human adenovirus type 5 backbone by homologous recombination in BJ5183 *E. coli* cells. The Ad vectors were produced in ProVector™ cells (Sirion Biotech GmbH, Germany) and purified using a CsCl gradient as described [20]. For titration ProVector™ cells were infected for 46 h, fixed and virus plaques stained using an anti-adenovirus hexon protein antibody (Santa Cruz Biotechnology) followed by a horseradish peroxidase (HRP)-conjugated goat anti-mouse immunoglobulin antibody (Dako) and 3,3′-diaminobenzidine substrate. The infectious units (IFU) per mL were determined by counting stained virus plaques.

The MVA-SIV vector encoding SIVmac239 Gag, Pol and Env (truncated at aa 733) was kindly provided by Dr. Patricia L. Earl (National Institutes of Health (NIH), USA). The production and titration of MVA vectors was carried out as described elsewhere [21].

### In vitro characterization of Ad-HIVB

#### VLP purification and Western Blot

Vero cells were seeded and infected with 50 IFU/cell Ad-HIVB. 48 h post infection the supernatant was harvested and VLPs were purified and concentrated to 160× the original concentration as described [22].

The Gag-P2A protein was detected in VLP samples by Western Blot using an anti-2A peptide antibody (Millipore) followed by an HRP-conjugated goat anti-rabbit Ig antibody. The membrane was developed using LumiGLO Peroxidase Chemiluminescent Substrate (KPL, 54-61-00) and the signal detected in an ImageQuant LAS 4000 (GE Healthcare Life Sciences).

#### Cell surface expression analysis

Env surface expression on Vero cells was analyzed 2 days post infection with 50 IFU/cell Ad-HIVB. The cells were stained with the monoclonal antibody clones VRC01 [NIH AIDS Reagent Program (NARP)], PGT145 [International AIDS Vaccine Initiative (IAVI)] and PGT151 (IAVI). Binding of the antibodies was detected using anti-human IgG Fc-APC antibody (BioLegend, 409305) and the fluorescence of the cells acquired in an LSRII instrument (BD Biosciences). The data was analyzed with FlowJo 10 software (Tree Star, Ashland, OR).

### Immunizations

Table 1 shows which viruses, IFUs and injection sites were used for the immunizations in the different experiments. Vaccines were injected intramuscularly in a total volume of 50 µL PBS under isoflurane anaesthesia.

**Table 1:** Summary of all immunization regimens showing the times of immunization (with viruses, IFUs and injections sites used), bleeding to obtain serum samples to assess antibody responses and intracellular cytokine staining (ICS) for the analysis of T-cell responses

Experiment	Group	Time	Intervention	Virus	IFU/mouse	Injection site
Immunogenicity of Ad-HIVB	–	Day 0	Immunization	Ad-HIVB	1 × 10^8^	Lower right leg
		Day 18	ICS			
Immunogenicity of Ad-Ii-SIVCErvv	–	Day 0	Immunization	Ad-Ii-SIVCErvv	2 × 10^8^	Lower right leg
		Day 14	ICS			
Heterologous HIV-prime SIV-boost	without Ad-Ii-SIVCErvv	Day 0	Prime immunization	Ad-HIVB	1 × 10^8^	Lower right leg
		Day 50–57	Bleeding			
		Day 63–74	Boost immunization	MVA-SIV	5 × 10^7^	Lower right leg
		10 days post boost	Bleeding & ICS			
	with Ad-Ii-SIVCErvv	Day 0	Prime immunization	Ad-HIVB	1 × 10^8^	Lower right leg
				Ad-Ii-SIVCErvv	1 × 10^8^	Lower left leg
		Day 50–57	Bleeding			
		Day 63–74	Boost immunization	MVA-SIV	5 × 10^7^	Lower right leg
		10 days post boost	Bleeding & ICS			
Homologous SIV-prime SIV-boost	without Ad-Ii-SIVCErvv	Day 0	Prime immunization	Ad-SIV	2 × 10^8^	Upper right leg
		Day 56	Bleeding &			
			Boost immunization	MVA-SIV	1 × 10^7^	Upper left leg
		Day 311	Bleeding & ICS			
	with Ad-Ii-SIVCErvv	Day 0	Prime immunization	Ad-SIV	2 × 10^8^	Upper right leg
				Ad-Ii-SIVCErvv	2 × 10^8^	Upper left leg
		Day 56	Bleeding &			
			Boost immunization	MVA-SIV	1 × 10^7^	Upper left leg
		Day 311	Bleeding & ICS			

### Intracellular cytokine staining (ICS)

Mouse splenocytes were stimulated at 37 °C and 5% CO_2_ for 5 h with relevant peptide pools at 1 ng/µL (test of Ad-Ii-SIVCErvv, homologous prime-boost) or 0.67 ng/µL (test of Ad-HIVB, heterologous prime-boost) and 3 µM Monensin. Peptides were obtained from the NARP and the SIV CE peptide pool was constructed as described [11]. Subsequently, cells were stained according to standard protocols [23, 24] using anti-mouse antibodies (Biolegend): PerCP/Cy5.5-CD8, FITC-CD4, Pacific Blue™-B220, APC/Cy7-CD44, APC-IFNγ, PE/Cy7-TNFα. Flow cytometry was performed in an LSRII instrument (BD Biosciences) and the data analyzed with FlowJo 10 software (Tree Star, Ashland, OR) using the gating strategy shown in Additional file 1.

### Antibody response measurements

Antibody responses against the Env protein from SIVmac239, HIV-1 clade B and HIV-1 clade C were measured by enzyme linked immunosorbent assay (ELISA) as described [11]. Env was produced from the HIV-1 clade B clone BaL.26 (NARP), clade C clone Du172.17 SVPC4 (NARP) and SIVmac239 (cloned by Anne-Marie C. Andersson). Antibody titers in the homologous prime-boost experiment were calculated as the highest dilution factor of the serum with an absorbance exceeding the mean absorbance using serum from unvaccinated control animals + 3 standard deviations. The area under the curve was calculated from the absorbance curve using GraphPad Prism 7 software. For the avidity index the titer obtained with sodium citrate treatment was divided by the titer obtained without sodium citrate treatment. For analyzing the isotype of the induced antibodies, the serum dilution, which resulted in an absorbance of 2.0 in the previous ELISA, was used and isotype specific binding was measured in duplicates. HRP-linked goat anti-mouse secondary antibodies for IgG2a, IgG2b, IgG2c and IgG3 (Thermo Fisher Scientific) were used.

### Statistical analysis

Two-tailed, unpaired Mann–Whitney U tests were performed to compare immune responses between 2 groups or responses to 2 different peptide pools in one group. Significances are indicated by asterisks: * p ≤ 0.05; ** p ≤ 0.01; *** p ≤ 0.001. To assess correlations Spearman correlation was used followed by adjustment of p-values by the Holm–Sidak method. Statistical analyses were performed using GraphPad Prism 7 software.

## Results

### Vaccine design and characterization

#### Design of VLVs

**Fig. 1:**
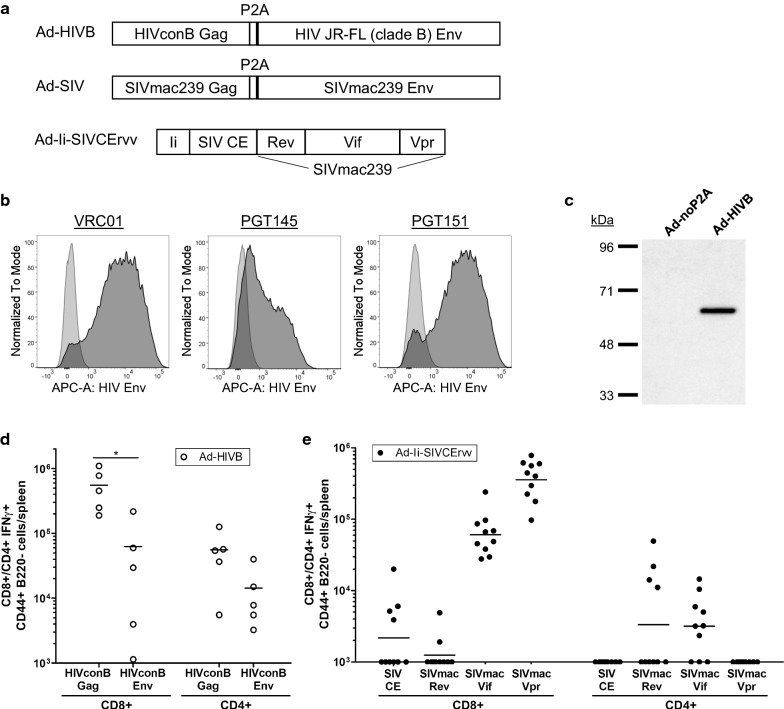
Design of adenoviral vectors and characterization in vitro and in vivo. **a** Schematic representation of the adenoviral vectors used in the study: Ad-HIVB encodes HIV-1 consensus clade B (HIVconB) Gag, P2A preceded by a glycine-serine-glycine linker (GSG; not noted in the figure) and HIV-1 JR-FL (clade B) Env; Ad-SIV encodes SIVmac239 Gag and Env separated by GSG and P2A; Ad-Ii-SIVCErvv encodes mouse Ii aa 1–75 (Ii) fused to SIV CEs and SIVmac239 Rev, Vif and Vpr. **b** Vero cells were infected with 50 IFU/cell Ad-HIVB and were stained after 2 days with the monoclonal antibodies VRC01, PGT145 and PGT151 targeting HIV-1 Env (shown in dark grey). APC-labelled anti-human IgG Fc served as a secondary antibody and the cells were analyzed by flow cytometry. Uninfected stained cells served as a control (light grey). **c** Vero cells were infected with 50 IFU/cell Ad-HIVB and after 48 h VLPs were purified from the cell culture supernatant. The VLP samples were analyzed by SDS-PAGE followed by Western Blot, which was stained with an anti-P2A antibody. Cells infected with an Ad5 vector not encoding P2A (Ad-noP2A) served as a negative control. **d** CD1 mice were vaccinated with Ad-HIVB (n = 5) and the induced T-cell responses were analyzed 18 days later by intracellular cytokine staining of stimulated splenocytes. The peptide pools used for stimulation are noted on the X-axis. The total numbers of IFNγ+ CD8+ and CD4+ T-cells per spleen were measured by flow cytometry. Horizontal lines indicate the geometric mean and significant differences are marked by asterisks with *(p ≤ 0.05). **e** CD1 mice were immunized with Ad-Ii-SIVCErvv (n = 10). After 14 days CD8+ and CD4+ T-cell responses to the vaccine antigens were analyzed as in **d**

We aimed to overcome the Env dominance in the heterologous Gag/homologous Env prime boost regimen in Andersson and Holst [11] by using both heterologous Gag and Env in prime and boost. Therefore, we designed a new Ad VLV encoding HIVconB Gag and HIV-1 JR-FL (clade B) Env (Ad-HIVB; Fig. 1a) to combine it with the MVA boost encoding SIVmac239 Gag, Pol and Env (MVA-SIV) in mice [11]. For the homologous Gag/Env prime-boost regimen we used the Ad-SIV (encoding SIVmac239 Gag and Env) and MVA-SIV VLVs from Andersson and Holst [11] (Fig. 1a). As the functionality of Ad-SIV had already been shown in the previous study, here we characterized only the Ad-HIVB VLV in vitro and in vivo.

#### Ad-HIVB expresses the encoded antigens and is immunogenic in mice

Ad-HIVB-infected Vero cells were stained for HIV-1 Env with three different broadly neutralizing monoclonal antibodies (VRC01, PGT145, PGT151). Analysis by flow cytometry confirmed cell surface expression of Env (Fig. 1b). We further assessed VLP formation capability of the VLV by Western Blot analysis of VLPs purified from the media of Ad-HIVB-infected Vero cells. In two independent experiments Gag-P2A was detectable with the expected band size of approx. 58 kDa (Fig. 1c).

To analyze the immunogenicity of the construct we vaccinated 5 CD1 mice with Ad-HIVB and measured the vaccine-induced T-cell responses after 18 days by ICS followed by flow cytometry (Fig. 1d). Ad-HIVB induced high numbers of HIVconB Gag-specific CD8+ T-cells, which were significantly higher than the HIVconB Env-specific CD8+ T-cell responses. CD4+ T-cell responses to HIVconB Gag were comparably high, taking into account that less CD4+ than CD8+ T-cells are found in the spleen. The number of Env-specific CD4+ T-cells was slightly lower than for Gag. These results confirm antigen-expression and VLP-formation by Ad-HIVB in cells and its immunogenicity in mice.

#### Ad-Ii-SIVCErvv induces T-cell responses to the accessory antigens

We aimed to further improve on the heterologous and homologous prime-boost regimen by inducing CD8+ T-cell responses to accessory antigens for acute control of infection and cross-reactive CE-specific CD8+ T-cell responses. To this end, we constructed a separate adenoviral vector encoding a fragment of the mouse Ii T-cell adjuvant, SIV CEs and SIVmac239 subdominant accessory proteins Rev, Vif and Vpr (Ad-Ii-SIVCErvv) as one fusion protein (Fig. 1a). 10 CD1 mice were vaccinated with Ad-Ii-SIVCErvv and the T-cell responses analyzed by ICS after 14 days (Fig. 1e). Few, low CD8+ T-cell responses could be measured to SIV CE and SIVmac239 Rev, but all animals showed high numbers of Vif- and Vpr-specific CD8+ T-cells. CD4+ T-cell responses to Rev were only found in a few mice in high numbers while Vif-specific CD4+ T-cell responses were induced in most animals. This shows that Ad-Ii-SIVCErvv raises T-cell responses to the encoded antigens in outbred mice.

### Heterologous HIV-prime SIV-boost regimen

For an experiment with heterologous Gag and Env in prime and boost we vaccinated 4 groups of mice. Two groups received only the priming vectors Ad-HIVB or Ad-HIVB together with Ad-Ii-SIVCErvv, the other two groups an additional MVA-SIV boost approx. 9–10 weeks after the prime. 10 days later we took serum samples to assess antibody responses and performed an ICS on splenocytes to analyze T-cell responses. In this time frame after MVA boosting the highest T-cell responses were expected as well as prominent antibody responses [25, 26]. In addition to peptide pools corresponding to the vaccine antigens (except Rev, Vif and Vpr), we used a peptide pool for SIVagm vervet (SIVagm) Gag to test the breadth and cross-reactivity of the induced Gag-specific T-cell responses. The sequence difference/similarity is roughly the same between HIVconB, SIVmac239 and SIVagm Gag [11].

#### CD8+ T-cell responses induced by heterologous HIV-prime SIV-boost regimen

**Fig. 2:**
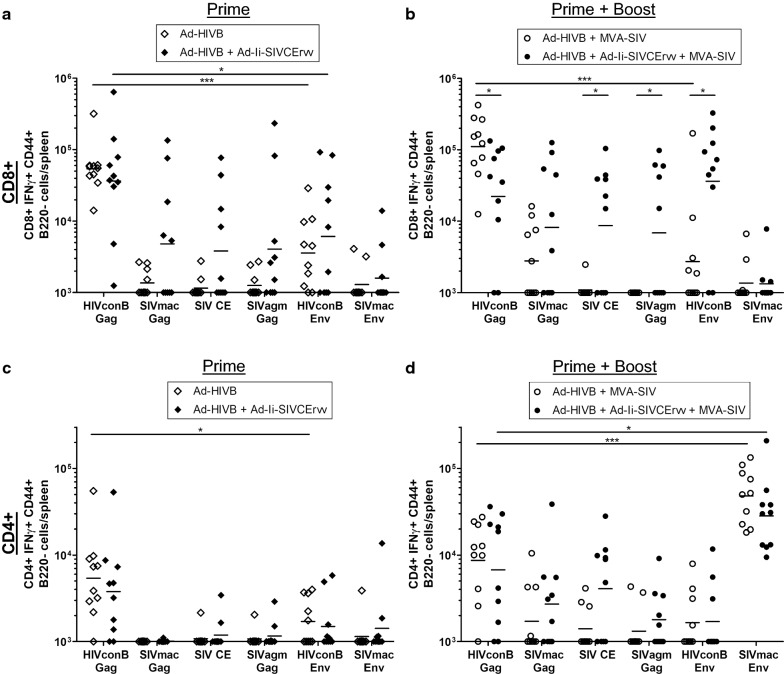
CD8+ and CD4+ T-cell responses elicited by heterologous HIV-prime SIV-boost regimen. We vaccinated CD1 mice with **a**, **c** only the priming vectors Ad-HIVB or Ad-HIVB together Ad-Ii-SIVCErvv or **b**, **d** boosted them with MVA-SIV approx. 9–10 weeks after the prime (n = 10 for each group). 10 days after the boost we measured the numbers of IFNγ+ CD8+ (**a**, **b**) and CD4+ (**c**, **d**) T-cells per spleen by stimulating splenocytes with the noted peptide pools and analyzing them by intracellular cytokine staining followed by flow cytometry. Horizontal lines mark the geometric mean and significant differences are indicated by asterisks with *(p ≤ 0.05), **(p ≤ 0.01) and ***(p ≤ 0.001)

In the two groups receiving only the priming vector Ad-HIVB with or without Ad-Ii-SIVCErvv, no significant differences were detectable in CD8+ T-cell responses towards Gag or Env epitopes (Fig. 2a). The CD8+ T-cell response to HIVconB Gag was significantly stronger than to HIVconB Env in both groups and the Ad-Ii-SIVCErvv group showed slightly broader responses to the different Gags and SIV CE. Including the MVA-SIV boost, HIVconB Gag-specific CD8+ T-cell responses were significantly higher in the group without Ad-Ii-SIVCErvv, while the SIV CE- and SIVagm Gag-specific responses were significantly higher in the Ad-Ii-SIVCErvv group (Fig. 2b). In the boosted Ad-Ii-SIVCErvv group we observed significantly more HIVconB Env-specific CD8+ T-cells than without the boost (p = 0.0468), which was not observed for the group without Ad-Ii-SIVCErvv. As a result, in the group without Ad-Ii-SIVCErvv, CD8+ T-cell responses to HIVconB Gag were significantly higher than to HIVconB Env. Accordingly, the completely heterologous prime-boost regimen could overcome the immunodominance of Env that has been observed when only varying Gag [11], but it could not select for cross-reactive or CE-specific CD8+ T-cell responses. In combination with Ad-Ii-SIVCErvv the CD8+ T-cell responses to HIVconB Gag were even slightly lower than to HIVconB Env, indicating a reappearance of Env dominance. However, addition of Ad-Ii-SIVCErvv to the vaccination regimen increased the breadth of CD8+ T-cell responses to the different Gags tested, which could potentially be explained by the included CEs priming T-cell specificities with broadened recognition of Gag sequences.

#### CD4+ T-cell responses induced by the heterologous HIV-prime SIV-boost regimen

Without the boost, the highest CD4+ T-cell responses were induced to HIVconB Gag. In the group without Ad-Ii-SIVCErvv these responses were significantly higher than HIVconB Env-specific CD4+ T-cell responses (Fig. 2c).

Boosting vaccination with MVA-SIV had only a moderate effect on most specificities but significantly increased the CD4+ T-cell responses to SIV CE in the Ad-Ii-SIVCErvv group (p = 0.0251) and SIVmac239 Env in both groups (p < 0.001) (Fig. 2d). This resulted in CD4+ T-cell responses dominated by SIVmac239 Env in both groups and slightly broader responses to the Gags/CE in the Ad-Ii-SIVCErvv group.

#### Heterologous HIV-prime SIV-boost regimen induced broad Env-specific antibody responses

**Fig. 3:**
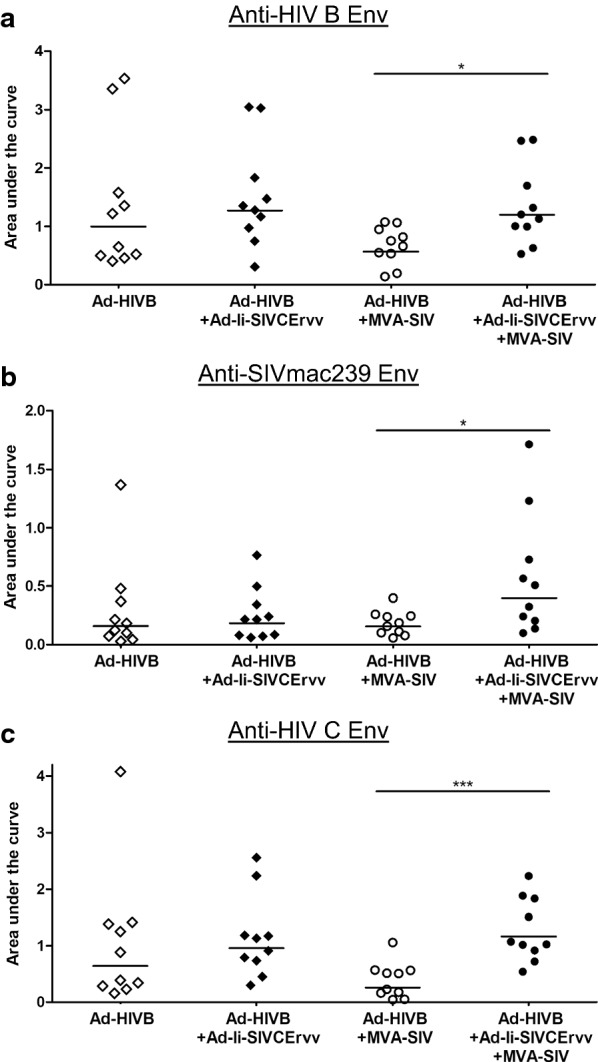
Heterologous HIV-prime SIV-boost regimen induced broadly reactive Env-specific antibody responses. Serum samples were taken 10 days after the boost and analyzed for antibody binding to lyzed pseudoviruses carrying **a** HIV-1 clade B (HIV B), **b** SIVmac239 and **c** HIV-1 clade C (HIV C) Env, by ELISA. Horizontal lines indicate the geometric mean and significant differences are marked by asterisks with *(p ≤ 0.05), **(p ≤ 0.01) and ***(p ≤ 0.001)

Vaccine-induced antibody responses to the 2 Env protein trimers used for immunizations (SIVmac239 and HIV-1 clade B Env) and additionally to HIV-1 clade C, to test cross-clade-reactivity, were tested by ELISA (Fig. 3). In all 4 vaccination groups cross-reactive antibody responses to Env were elicited, indicating broad lentivirus-specific responses induced by the VLVs. Boosting with MVA-SIV had little effect, but in combination with Ad-Ii-SIVCErvv in the prime yielded responses against all 3 tested Envs that were significantly higher than in the boosted group without Ad-Ii-SIVCErvv.

#### Correlations of immune responses induced by heterologous HIV-prime SIV-boost regimen

The T-cell and antibody responses measured in the boosted groups were analyzed for correlations using Spearman correlation and the obtained p-values were adjusted for multiple comparisons using the Holm–Sidak method.

Without Ad-Ii-SIVCErvv the SIVmac239 Gag- and HIVconB Env-specific CD8+ T-cell responses as well as antibody responses to SIVmac Env and HIV-1 clade C Env correlated significantly (Table 2a). In the Ad-Ii-SIVCErvv-vaccinated group CD4+ T-cell responses to each of the following peptide pools were significantly correlated with the CD8+ T-cell response to the same pool: HIVconB Gag, SIVmac239 Gag, SIV CE, SIVagm Gag, HIVconB Env and SIVmac239 Env (Table 2b). Besides, CD4+ and CD8+ T-cell responses to SIVmac239 Gag correlated significantly with CD4+ and CD8+ T-cell responses to SIVagm Gag. Antibody responses to HIV-1 clade B Env and HIV-1 clade C Env were correlated.

**Table 2:** Correlation of T-cell and antibody responses in the heterologous HIV-prime SIV-boost regimen

*a* Ad-HIVB +MVA-SIV	CD4+ T-cells						CD8+ T-cells						Antibodies		
	HIV conB Gag	SIV mac Gag	SIV CE	SIV agm Gag	HIV conB Env	SIV mac Env	HIV conB Gag	SIV mac Gag	SIV CE	SIV agm Gag	HIV conB Env	SIV mac Env	anti-HIV B Env	anti-SIV mac Env	anti-HIV C Env
**CD4+ T-cells**															
HIVconB Gag															
SIVmac Gag	0.479														
SIV CE	0.333	0.292													
SIVagm Gag	0.022	0.533	0.689												
HIVconB Env	0.019	0.673	0.275	0.044											
SIVmac Env	0.096	0.977	0.400	0.044	0.098										
**CD8+ T-cells**															
HIVconB Gag	0.233	0.453	0.306	0.800	0.648	0.513									
SIVmac Gag	0.043	0.984	0.785	0.400	0.211	0.072	0.023								
SIV CE	0.600	0.900	0.100	1.000	0.200	0.800	0.600	0.800							
SIVagm Gag															
HIVconB Env	0.007	1.000	0.253	0.044	0.012	0.017	0.072	*0.002*	0.200						
SIVmac Env	0.931	0.333	0.067	0.611	1.000	1.000	0.214	0.500	0.200		0.722				
**Antibodies**															
anti-HIV B Env	0.330	0.274	0.025	0.956	0.442	0.427	0.013	0.159	0.200		0.187	0.008			
anti-SIVmac Env	0.973	0.794	0.531	0.867	0.556	0.233	0.407	0.246	0.600		0.335	0.039	0.096		
anti-HIV C Env	0.707	0.962	0.264	0.911	0.383	0.387	0.096	0.199	0.400		0.230	0.031	0.015	*0.001*	

*b* Ad-HIVB + Ad-Ii-SIVCErvv +MVA-SIV	CD4+ T-cells						CD8+ T-cells						Antibodies		
	HIV conB Gag	SIV mac Gag	SIV CE	SIV agm Gag	HIV conB Env	SIV mac Env	HIV conB Gag	SIV mac Gag	SIV CE	SIV agm Gag	HIV conB Env	SIV mac Env	anti-HIV B Env	anti-SIV mac Env	anti-HIV C Env
**CD4+ T-cells**															
HIVconB Gag															
SIVmac Gag	0.110														
SIV CE	0.300	0.006													
SIVagm Gag	0.064	*2.6E−04*	0.010												
HIVconB Env	0.645	0.010	0.024	0.042											
SIVmac Env	0.042	0.599	0.475	0.639	1.000										
**CD8+ T-cells**															
HIVconB Gag	*5.5E−07*	0.110	0.300	0.064	0.645	0.042									
SIVmac Gag	0.110	*6.6E−06*	0.006	*2.6E−04*	0.010	0.599	0.110								
SIV CE	0.300	0.006	*6.6E−06*	0.010	0.024	0.475	0.300	0.006							
SIVagm Gag	0.064	*2.6E−04*	0.010	*3.3E−05*	0.042	0.639	0.064	*2.6E−04*	0.010						
HIVconB Env	0.645	0.010	0.024	0.042	*5.5E−07*	1.000	0.645	0.010	0.024	0.042					
SIVmac Env	0.042	0.599	0.475	0.639	1.000	*0.001*	0.042	0.599	0.475	0.639	1.000				
**Antibodies**															
anti-HIV B Env	0.075	0.299	0.122	0.215	0.462	0.008	0.075	0.299	0.122	0.215	0.462	0.008			
anti-SIVmac Env	0.168	0.205	0.552	0.316	0.162	0.156	0.168	0.205	0.552	0.316	0.162	0.156	0.096		
anti-HIV C Env	0.125	0.528	0.375	0.394	0.399	0.039	0.125	0.528	0.375	0.394	0.399	0.039	*0.002*	0.031	

p-values of Spearman correlation in MVA-SIV-boosted groups *a* without Ad-Ii-SIVCErvv and *b* with Ad-Ii-SIVCErvv. p-values that remained significant after adjustment with the Holm–Sidak-method are marked in italic

### Homologous SIV-prime SIV-boost regimen

In addition to optimizing the prime-boost regimen from Andersson and Holst by using heterologous Env, we also tested if we could improve on the prime-boost regimen from Andersson and Holst with homologous SIVmac239 Gag and Env by adding Ad-Ii-SIVCErvv [11]. Therefore, we vaccinated 2 groups of mice with the Ad-SIV prime with one group receiving the additional priming vector Ad-Ii-SIVCErvv. The mice were boosted with MVA-SIV 8 weeks after the prime and the T-cell and antibody responses were analyzed 8 months after the boost. Andersson and Holst had already analyzed the acute responses following homologous prime-boost immunization and our results in the heterologous prime-boost indicated that the Ad-Ii-SIVCErvv vaccine could alter antibody responses. Therefore, we chose a late time point after vaccination to assess the effect of Ad-Ii-SIVCErvv on the memory T-cell and antibody responses induced by the vaccines. All peptide pools used in this study were from SIVmac239.

#### CD8+ T-cell responses induced by homologous SIV-prime SIV-boost regimen

**Fig. 4:**
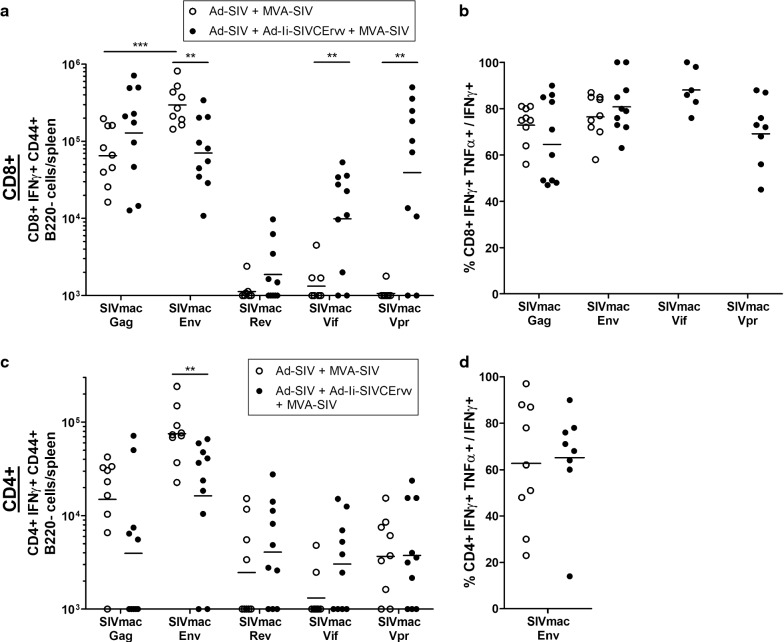
CD8+ and CD4+ T-cell responses induced by homologous SIV-prime SIV-boost regimen. CD1 mice were immunized with the priming vectors Ad-SIV (n = 9) or Ad-SIV and Ad-Ii-SIVCErvv (n = 10) and boosted with MVA-SIV 8 weeks after the prime. 10 days later CD8+ and CD4+ T-cell responses were measured by stimulating splenocytes with the noted SIVmac239 peptide pools and analyzing them by intracellular cytokine staining followed by flow cytometry. **a**, **c** show the total numbers of IFNγ+ CD8+ (**a**) and CD4+ (**b**) T-cells per spleen and **b**, **d** the percentage of IFNγ+ TNFα+ CD8+/CD4+ T-cells of IFNγ+ CD8+/CD4+ T-cells for samples with more than 10^4 ^IFNγ+ CD8+ T-cells. Horizontal lines mark the geometric mean and significant differences are indicated by asterisks with *(p ≤ 0.05), **(p ≤ 0.01) and ***(p ≤ 0.001)

High Gag- and Env-specific CD8+ T-cell responses were present in both groups, but responses to Gag were higher when Ad-Ii-SIVCErvv was administered additionally (Fig. 4a). Env-specific CD8+ T-cell responses in the group without Ad-Ii-SIVCErvv were significantly stronger than Gag-specific responses in the same group and Env-specific responses in the Ad-Ii-SIVCErvv-vaccinated group. As a result, the ratio of Gag- to Env-specific CD8+ T-cells was significantly higher in the Ad-Ii-SIVCErvv group (p = 0.0021). In the Ad-Ii-SIVCErvv group some low CD8+ T-cell responses were detectable to Rev and moderate and comparably high responses to Vif and Vpr, with the latter 2 being significantly higher than the responses to these antigens in the group without Ad-Ii-SIVCErvv.

To evaluate the functionality of the vaccine-induced memory CD8+ T-cells we analyzed which percentage of IFNγ+ CD8+ T-cells simultaneously produced TNFα (Fig. 4b). Only samples with more than 10^4^ IFNγ+ CD8+ T-cells per spleen were considered, which was the case for all Gag- and Env-specific CD8+ T-cell responses and for the majority of Vif- and Vpr-specific CD8+ T-cell responses. Nearly all mice showed more than 50% double-positive cells for the tested vaccine antigens with geometric means between 65% and 85%, indicating that the quality of induced CD8+ T-cells was good and did not differ distinctly between groups.

#### CD4+ T-cell responses induced by homologous SIV-prime SIV-boost regimen

When mice were vaccinated with the homologous prime-boost without Ad-Ii-SIVCErvv, higher numbers of CD4+ T-cells reacted to Gag and significantly more CD4+ T-cells reacted to Env than in mice vaccinated with the additional prime (Fig. 4c). In the group without Ad-Ii-SIVCErvv, background CD4+ T-cell responses to the accessory antigens were observed and addition of Ad-Ii-SIVCErvv had only a remote effect on CD4+ T-cell responses towards Rev and Vif. Furthermore, responses to Vpr were not affected by including Ad-Ii-SIVCErvv indicating variation around the background response levels in the assay.

The percentage of IFNγ TNFα double-positive cells for Env-specific CD4+ T-cells was consistently positive, with a geometric mean above 60% for both groups and no detectable difference (Fig. 4d).

#### Antibody responses induced by homologous SIV-prime SIV-boost regimen

**Fig. 5:**
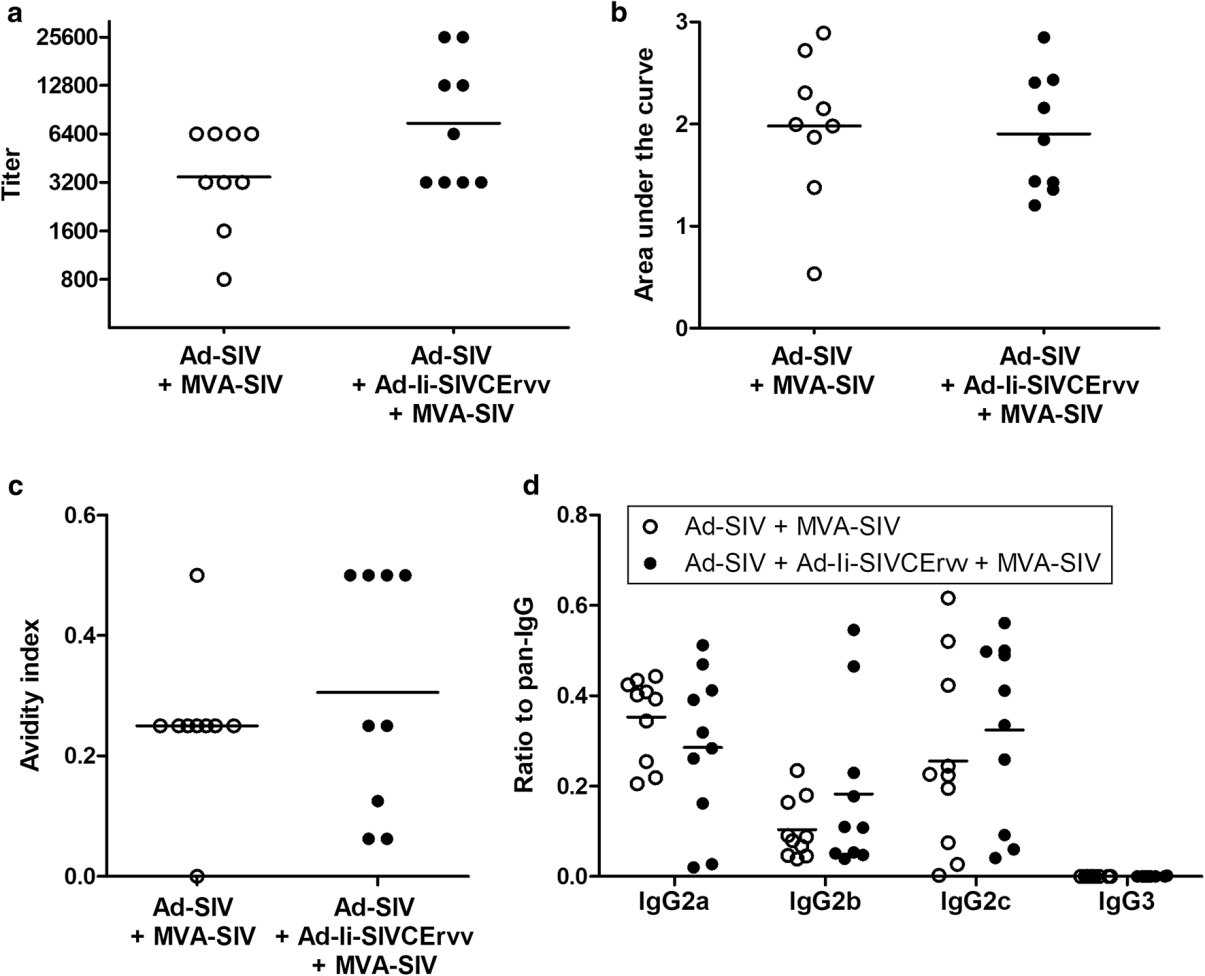
Addition of Ad-Ii-SIVCErvv to homologous SIV-prime SIV-boost regimen did not impact Env-specific antibody responses. Serum samples were taken at the time of the intracellular cytokine staining and analyzed for antibody binding to lyzed pseudoviruses carrying SIVmac239 Env by ELISA. **a** shows the antibody responses as titers and **b** as the AUC. In **c** the avidity index is depicted and in **d** the antibody responses detected with anti-IgG isotype-specific antibodies as the ratio to the not isotype-specific antibody (pan-IgG). Horizontal lines indicate the geometric mean, except in **c** where it depicts the mean because of a single mouse not having a measurable avidity index

In order to assess antibody responses, we calculated the titer as well as the area under the curve (AUC), which both did not differ significantly between the groups (Fig. 5a, b). In addition, the avidity of antibodies did not differ between the groups (Fig. 5c).

The induction of different IgG isotypes was further analyzed and is depicted in Fig. 5d as the AUC ratio to whole IgG serum antibodies detected using an isotype-unspecific pan-IgG secondary antibody. The ratio of IgG2a antibodies to total IgG or IgG1 has been proposed as a functional association with protective antibodies in some models in inbred mice, however, it was quite evident that CD1 mice were heterogenous for IgG2a and IgG2c, which made it impossible to produce meaningful ratios including either isotype. Nevertheless, more IgG2a and IgG2c than IgG2b were induced with no significant differences between the groups while no IgG3 was detected.

#### Correlation of immune responses induced by homologous SIV-prime SIV-boost regimen

In the group without Ad-Ii-SIVCErvv the AUC and titer of antibody responses correlated significantly (Table 3a). In the group with Ad-Ii-SIVCErvv, Env- and Vpr-specific CD4+ T-cell responses showed significant correlation (Table 3b). In addition, both the AUC and titer of the antibody responses correlated significantly with the avidity index.

**Table 3:** Correlation of T-cell and antibody responses in the homologous SIV-prime SIV-boost regimen

*a* Ad-SIV + MVA-SIV	CD4+ T-cells					CD8+ T-cells					Antibodies		
	Gag	Env	Rev	Vif	Vpr	Gag	Env	Rev	Vif	Vpr	AUC	Titer	Avidity
**CD4+ T-cells**													
Gag													
Env	0.086												
Rev	0.085	0.834											
Vif	0.694	0.250	0.792										
Vpr	0.262	0.731	0.342	0.444									
**CD8+ T-cells**													
Gag	0.678	1.018	0.151	0.500	0.363								
Env	0.437	0.410	0.611	0.889	0.082	0.017							
Rev	0.516	0.131	0.960	0.583	0.187	0.226	0.226						
Vif	0.476	0.901	0.181	1.000	0.782	0.615	0.460	0.940					
Vpr	0.667	1.111	0.889	0.222	0.889	0.667	0.444	1.000	0.333				
**Antibodies**													
AUC	0.097	0.776	0.228	0.944	0.853	0.644	0.948	0.583	0.226	0.444			
Titer	0.263	0.630	0.337	0.889	0.673	0.616	0.913	0.230	0.480	0.667	*0.001*		
Avidity	0.083	1.000	0.139	1.583	0.056	0.583	0.778	1.417	1.417	1.778	1.000	0.750	

*b* Ad-SIV + Ad-Ii-SIVCErvv + MVA-SIV	CD4+ T-cells					CD8+ T-cells					Antibodies		
	Gag	Env	Rev	Vif	Vpr	Gag	Env	Rev	Vif	Vpr	AUC	Titer	Avidity
**CD4+ T-cells**													
Gag													
Env	0.046												
Rev	0.629	0.015											
Vif	0.182	0.223	0.079										
Vpr	0.014	*0.002*	0.074	0.111									
**CD8+ T-cells**													
Gag	0.461	0.496	0.463	0.459	0.862								
Env	0.296	0.106	0.256	0.866	0.305	0.081							
Rev	0.503	0.777	0.430	0.150	0.307	0.125	0.828						
Vif	0.665	0.909	0.748	0.955	0.456	0.911	0.306	0.763					
Vpr	0.116	0.480	0.896	1.002	0.082	0.220	0.845	0.144	0.199				
**Antibodies**													
AUC	0.075	0.164	0.515	0.477	0.221	0.843	0.230	0.422	0.389	0.853			
Titer	0.040	0.157	0.558	0.272	0.085	0.738	0.629	0.725	0.410	0.512	0.004		
Avidity	0.053	0.112	0.458	0.346	0.127	0.804	0.601	0.495	0.798	0.798	*0.002*	*0.002*	

p-values of Spearman correlation in MVA-SIV-boosted groups *a* without Ad-Ii-SIVCErvv and *b* with Ad-Ii-SIVCErvv. p-values that remained significant after adjustment with the Holm–Sidak-method are marked in italic

## Discussion

To induce protective B-cell responses and avoid the dominance of non-protective Env-specific T-cell responses observed in Andersson and Holst [11], we tested an immunization regimen using both heterologous Gag and Env in prime and boost. Therefore, we constructed a VLV (Ad-HIVB) encoding HIVconB Gag and HIV-1 JR-FL Env (clade B). We confirmed antigen expression and VLP formation in vitro and the immunogenicity of the vector in vivo (Fig. 1a–d).

As a second goal we aimed to focus the CD8+ T-cell response on putatively more protective Gag CEs and to induce T-cell responses to accessory antigens which have been shown to improve early control of infection [15, 17, 18]. An adenovirus encoding a fragment of the Ii, SIV CEs, SIVmac239 Rev, Vif and Vpr (Ad-Ii-SIVCErvv; Fig. 1a) was used as an additional prime in the heterologous as well as homologous vaccination regimen from Andersson and Holst. Immunization of outbred CD1 mice with this vector alone induced few CD8+ T-cell responses to SIV CE, strong CD8+ T-cell responses to Vif and Vpr and some animals raised high numbers CD4+ T-cells to Rev and most to Vif (Fig. 1e). We hypothesized that, even though priming of SIV CE-specific T-cell responses was not very strong with this vector, they could be boosted by full-length Gag encoded in MVA-SIV and that even minor T-cell responses towards the CEs might influence response to Gag-containing VLPs.

### Heterologous HIV-prime SIV-boost experiment

In the heterologous prime-boost regimen the primes alone (Ad-HIVB or Ad-HIVB together with Ad-Ii-SIVCErvv) induced more HIVconB Gag- than Env-specific CD8+ T-cell responses (Fig. 2a). The MVA-SIV boosted group without Ad-Ii-SIVCErvv showed significantly higher numbers of HIVconB Gag- than HIVconB Env-specific CD8+ T-cells (Fig. 2b). This observation indicates that the completely heterologous prime-boost regimen successfully prevented the Env immunodominance of the partly heterologous regimen (heterologous Gag + homologous Env) observed in Andersson and Holst [11]. Considering solely the T-cell response, a better viral control should be achieved as lower viral loads have been shown to correlate directly with CD8+ T-cell responses targeting Gag and inversely with CD8+ T-cells specific for Env in humans [4, 5].

Unfortunately, the heterologous Gag/Env immunization regimen failed to raise the expected broadly reactive Gag-specific CD8+ T-cell responses since responses to SIV CE and SIVagm Gag were still low.

Notably, the heterologous immunization regimen in combination with Ad-Ii-SIVCErvv succeeded in raising broader Gag/CE-specific CD8+ and CD4+ T-cell responses than the immunization without Ad-Ii-SIVCErvv (Fig. 2b). These broad responses could be explained by the priming of SIV CE-specific T-cell responses by Ad-Ii-SIVCErvv, which were detectable in mice receiving only the prime, although they were not significantly higher than in the group without Ad-Ii-SIVCErvv. In the boosted Ad-Ii-SIVCErvv group SIV CE-specific responses were indeed slightly higher than in the group with only the prime which is in accordance with the results from Kulkarni et al. who found that CE-specific T-cell responses could be boosted by vaccination with full-length Gag [8].

In the Ad-Ii-SIVCErvv group CD4+ T-cell responses to one peptide pool correlated significantly with the CD8+ T-cell responses to the same pool (Table 2b), which was true for all peptide pools in this group. In contrast there was no correlation between CD4+ and CD8+ T-cell responses to the same antigen in the group without Ad-Ii-SIVCErvv (Table 2a). This suggests that the inclusion of Ad-Ii-SIVCErvv in the immunization regimen through an unknown mechanism influenced the CD4+ and CD8+ T-cell responses to the same antigen. These correlations led us to hypothesize that CD4+ T-cells might preferentially stimulate CD8+ T-cells with specificity to the same antigen in the boosted Ad-Ii-SIVCErvv group. Broader Gag/CE-specific CD4+ T-cell responses with higher magnitude were elicited compared to the group without Ad-Ii-SIVCErvv (Fig. 2d), although the differences were not statistically significant. If these would preferentially provide T-cell help to CD8+ T-cells with the same specificity, they could help boost the observed cross-reactive Gag/CE-specific CD8+ T-cell responses. The assumption that these CD8+ T-cell responses are indeed cross-reactive and do not merely result from different specificities, is supported by the strong correlation between SIVmac239 Gag-specific CD4+ and CD8+ T-cell responses and SIVagm Gag-specific CD4+ and CD8+ T-cell responses (Table 2b). Regarding solely the breadth of the response, the heterologous immunization regimen with Ad-Ii-SIVCErvv was clearly superior to the one without Ad-Ii-SIVCErvv.

In the MVA-boosted group receiving the additional Ad-Ii-SIVCErvv prime, Env dominance reappeared and slightly more HIVconB Env-specific than HIVconB Gag-specific CD8+ T-cells were detectable (Fig. 2b). The reason for this reappearance of Env immunodominance is not clear, but we speculate that it could result from differences in the CD4+ T-cell help. We could assume that because Gag/CE-specific CD8+ T-cell responses were lower, albeit more cross-reactive, in the Ad-Ii-SIVCErvv group compared to the group without Ad-Ii-SIVCErvv, they were not able to dominate over and inhibit the expansion of HIVconB Env-specific CD8+ T-cells. Additionally, CD4+ T-cell responses to the accessory antigens, which were not measured in this assay due to practical limitations in sample processing, could be responsible for the preferred boosting of HIVconB Env in the Ad-Ii-SIVCErvv group. Further mechanistic effects such as altered T-cell quality rather than quantity, lack of IFNγ production in T-cells or induction of regulatory T-cells might play a role as well.

In all groups broad antibody responses reacting to Env from 2 different HIV-1 clades were elicited and even showed some reactivity to SIVmac239 Env (Fig. 3). It is striking that we could induce such broadly reactive Env-binding antibodies that also bind to SIVmac239 Env. Naturally, we cannot be sure if cross-reactive antibodies are responsible for this effect or if we instead induced additional SIVmac239 Env-specific antibodies with the boost. HIV-1 Env-targeting monoclonal antibodies with cross-reactivity to chimpanzee SIV Env have been described previously in mice [27]. Therefore, it seems possible that we indeed detected HIV-1/SIV cross-reactive antibodies. Additionally, there is a strong positive correlation between antibody responses to SIVmac239 and HIV-1 clade C Env in the group without Ad-Ii-SIVCErvv, which could support this assumption (Table 2a).

Antibody responses to HIV-1 clade B, clade C and SIVmac239 Env were significantly higher in the Ad-Ii-SIVCErvv group combined with the MVA boost compared to the boosted group without Ad-Ii-SIVCErvv (Fig. 3). This could be explained by the higher numbers of Gag/CE-specific CD4+ T-cells in this group providing intrastructural help for Env-specific B-cells during the boost as previously described for VLP vaccines by Nabi et al. [14].

### Homologous SIV-prime SIV-boost regimen

In the homologous prime-boost regimen CD8+ T-cell responses to Env were higher than to Gag in the group without Ad-Ii-SIVCErvv 8 months after the boost (Fig. 4a). These results differ from the responses observed by Andersson and Holst 10 days after the boost [11], where stronger Gag- than Env-specific T-cell responses were measured. This could most likely be due to different kinetics in the Gag- and Env-specific CD8+ T-cell responses, with longer expansion and/or greater stability of Env-specific CD8+ T-cells.

Adding Ad-Ii-SIVCErvv to the vaccination regimen induced higher numbers of Gag- than Env-specific CD8+ T-cells (Fig. 4a). Thus, the ratio of Gag- to Env-specific CD8+ T-cells was significantly higher than in the group without Ad-Ii-SIVCErvv. A likely explanation is an effect of the encoded CEs since in Andersson and Holst broad Gag- and CE-specific CD8+ T-cell responses could be induced [11]. In Martins et al. slightly higher Gag- than Env-specific CD8+ T-cell responses were observed as well in an immunization regimen including Gag, Env, Vif, Rev, Tat, and Nef [17], which would suggest that the accessory antigens might play a role as well in this context. In an efficacy model the higher Gag- compared to Env-specific CD8+ T-cell responses in the Ad-Ii-SIVCErvv group would normally be predicted to result in better long-term viral control as CD8+ T-cells targeting Gag are correlated with a lower viral load [4, 5] and are mediating ex vivo control [28], while T-cell targeting of Env is correlated with a higher viral load [4].

In combination with the homologous prime-boost regimen Ad-Ii-SIVCErvv induced CD8+ T-cell responses to the accessory antigens with a similar pattern as the administration of this vector alone (Figs. 1e, 4a). Responses to Vpr were the most prominent followed by Vif, while in combination with the VLVs even Rev-specific CD8+ T-cell responses were detectable. The induction of accessory antigen-specific CD8+ T-cells is likely to improve early control of infection as shown in Xu et al., Hel et al. and Martins et al. [15, 17, 18].

In the group vaccinated with Ad-Ii-SIVCErvv, Gag- and Env-specific CD4+ T-cell responses were lower than in the group without Ad-Ii-SIVCErvv, while the responses to Rev and Vif were slightly higher (Fig. 4c). It is possible that, with the inclusion of the accessory antigens, antigen competition might be responsible for this shift as a similar phenomenon was also observed in Hel et al. [18]. When a Gag-Pol-Env and a Rev-Tat-Nef vaccine were combined, lower T-cell responses to the immunodominant antigens Gag, Env and Nef were measured compared to the responses to either of the 2 vaccines alone. However, Hel et al. did not distinguish between CD4+ and CD8+ T-cells and it was not clear if competition between the immunodominant antigens was responsible or if the subdominant antigens Rev and Tat played a role.

In the Ad-Ii-SIVCErvv-vaccinated group Env-specific CD4+ T-cell responses correlated significantly with Vpr-specific and almost significantly with Rev-specific CD4+ T-cells, suggesting that there might be interplay between these T-cell specificities (Table 3b). However, caution should be taken when interpreting the lower CD4+ T-cell responses as animals in the group without Ad-Ii-SIVCErvv occasionally exhibited low CD4+ T-cell responses to the accessory antigens after subtraction of the background values. Nevertheless, whatever the mechanism, the observed shift away from the structural antigens can be considered beneficial as it has been shown that CD4+ T-cell responses towards the structural antigens are associated with infection-enhancement [19]. In addition, CD4+ T-cells specific for Rev and Vif could stimulate the expansion of CD8+ T-cells with the same specificities and thus help to improve early viral control.

Ad-Ii-SIVCErvv did not influence the antibody responses, which were very similar in antibody titer/AUC, avidity and isotype distribution (Fig. 5).

In both groups more IgG2a and IgG2c than IgG2b and no IgG3 antibodies were induced. IgG2b is considered more important in the early infection, while IgG2a is more associated with antigen clearance through induction of e.g. antibody-dependent cellular cytotoxicity later [29]. However, in our case, the use of IgG2a levels as a proxy for antibody effector mechanisms were not very informative as the CD1 strain was found to be heterogeneous for IgG2a and IgG2c.

Altogether, we found that it was possible to include a subdominant antigen vaccine, modified from the one described in Xu et al. [15], in a vaccination regimen consisting of dominant antigens with expectedly beneficial effects for the T-cell responses in regard to the protection-associated responses. This effect can be considered synergistic as the addition of Ad-Ii-SIVCErvv not only raised immune responses to the accessory antigens, but also beneficially impacted the T-cell responses to Gag and Env.

In Hel et al. antigen competition could be observed when combining dominant and subdominant antigens [18]. As we do not have data on the immune response to Ad-Ii-SIVCErvv alone 10 months after the immunization, it is hard to say if the Gag/Env-vaccine impacted the T-cell response to the accessory antigens negatively. However, in combination with the VLVs we observed higher CD8+ T-cell responses to Rev than with the Ad-Ii-SIVCErvv-vaccination alone (compare Figs. 1e, 4a) and the Vpr-specific CD8+ T-cell responses matched the Env-specific ones in magnitude. Therefore, it is possible that the co-administration with the Gag/Env prime exerted a positive effect on the response to the accessory antigens.

## Conclusion

The fully heterologous Gag/Env VLV prime-boost regimen was able to overcome the Env immunodominance in the CD8+ T-cell responses observed in Andersson and Holst, which suggests a more protective response. However, the expected increased breadth of Gag-specific CD8+ T-cell responses was not obtained. In combination with an additional prime encoding SIV Gag CEs and accessory antigens, Env dominated the CD8+ T-cell response but broader Gag-specific CD8+ T-cell responses and higher levels of Env-binding antibodies were induced.

The homologous immunization regimen in combination with Ad-Ii-SIVCErvv was able to improve the induction of immune responses that have previously been correlated with protection against HIV-1 acquisition as compared to the VLVs alone. This includes Env-binding IgG antibodies, and T-cell responses associated with early (accessory antigen-specific CD8+ T-cells) and late viral control (more Gag- than Env-specific CD8+ T-cells, less Gag/Env-specific CD4+ T-cells). As this seems very promising, the next step would be to test this homologous immunization regimen in a macaque efficacy trial using SIVmac251 as the challenge strain.

## Additional file

**Additional file 1.** Gating strategy for flow cytometry analysis of intracellular cytokine staining. First, the cells were gated for single cells (**a**) in a side scatter (SSC)-A/SSC-W plot, which were further gated for the lymphocyte population (**b**) in a plot of forward scatter (FCS)-A and SSC-A. The lymphocyte population was gated for CD8+ B220- cells (**c**) and CD4+ B220- cells (**d**). Next, these cells were gated for IFNγ+ CD44+ cells (**e**, upper rectangle), here shown representatively for the CD8+ population. From these populations the absolute number of IFNγ+ CD44+ B220- CD8+ and CD4+ T-cells was calculated by multiplying the percentage of IFNγ+ CD44+ B220- CD8+/CD4+ cells of the lymphocytes with the number of counted lymphocytes per spleen. To obtain the percentage of double positive (IFNγ+ TNFα+) cells of IFNγ+ CD8+ and CD4+ T-cells, CD8+/CD4+ B220- cells were also gated for CD44+ cells (**e**, both rectangles) in homologous prime-boost regimen and subsequently for IFNγ- TNFα+ (**f**, left rectangle) and IFNγ+ TNFα+ (**f**, right rectangle) cells.

## Data Availability

All data generated and analyzed during this study are included in this published article and its additional file. Unique materials generated in the study are available for non-commercial purposes.

## Abbreviations

Ad: human adenovirus type 5
Ad-HIVB: Ad vector encoding HIVconB Gag and JR-FL Env
Ad-Ii-SIVCErvv: Ad vector encoding Ii, SIV p27 Gag CEs and SIVmac239 Rev, Vif and Vpr
Ad-SIV: Ad vector encoding SIVmac239 Gag and Env
CE: conserved element
ELISA: enzyme-linked immunosorbent assay
Env: envelope
Gag: group-specific antigen
HIV-1: human immunodeficiency virus type 1
HIVconB: HIV-1 consensus clade B
HRP: horseradish peroxidase
IAVI: International AIDS Vaccine Initiative
ICS: intracellular cytokine staining
IFNγ: interferon γ
IFU: infectious units
Ii: MHC class II-associated invariant chain
MVA: modified vaccinia virus Ankara
MVA-SIV: MVA vector encoding SIVmac239 Gag, Pol and Env
NARP: NIH AIDS Reagent Program
Nef: negative factor
NHP: non-human primates
NIH: National Institutes of Health
PBS: phosphate buffered saline
Pol: DNA polymerase
Rev: regulator of expression of virion proteins
SIV: simian immunodeficiency virus
SIVagm: SIVagm vervet
Tat: trans-activator of transcription
TNFα: tumor necrosis factor α
Vif: viral infectivity factor
VLP: virus-like particle
VLV: virus-like vaccine
Vpr: viral protein R
