# Supplementary material for: Virus-like vaccines against HIV/SIV synergize with a subdominant antigen T cell vaccine
Source: J Transl Med. 2019 May 24;17:175. doi: 10.1186/s12967-019-1924-1 (PMC6534914; doi:10.1186/s12967-019-1924-1)
Supplement: Supplementary file 1 — Additional file 1. Gating strategy for flow cytometry analysis of intracellular cytokine staining. First, the cells were gated for single cells (a) in a side scatter (SSC)-A/SSC-W plot, which were further gated for the lymphocyte population (b) in a plot of forward scatter (FCS)-A and SSC-A. The lymphocyte population was gated for CD8+ B220- cells (c) and CD4+ B220- cells (d). Next, these cells were gated for IFNγ+ CD44+ cells (e, upper rectangle), here shown representatively for the CD8+ population. From these populations the absolute number of IFNγ+ CD44+ B220- CD8+ and CD4+ T-cells was calculated by multiplying the percentage of IFNγ+ CD44+ B220- CD8+/CD4+ cells of the lymphocytes with the number of counted lymphocytes per spleen. To obtain the percentage of double positive (IFNγ+ TNFα+) cells of IFNγ+ CD8+ and CD4+ T-cells, CD8+/CD4+ B220- cells were also gated for CD44+ cells (e, both rectangles) in homologous prime-boost regimen and subsequently for IFNγ- TNFα+ (f, left rectangle) and IFNγ+ TNFα+ (f, right rectangle) cells. [file 12967_2019_1924_MOESM1_ESM.pptx]

## Slide 1
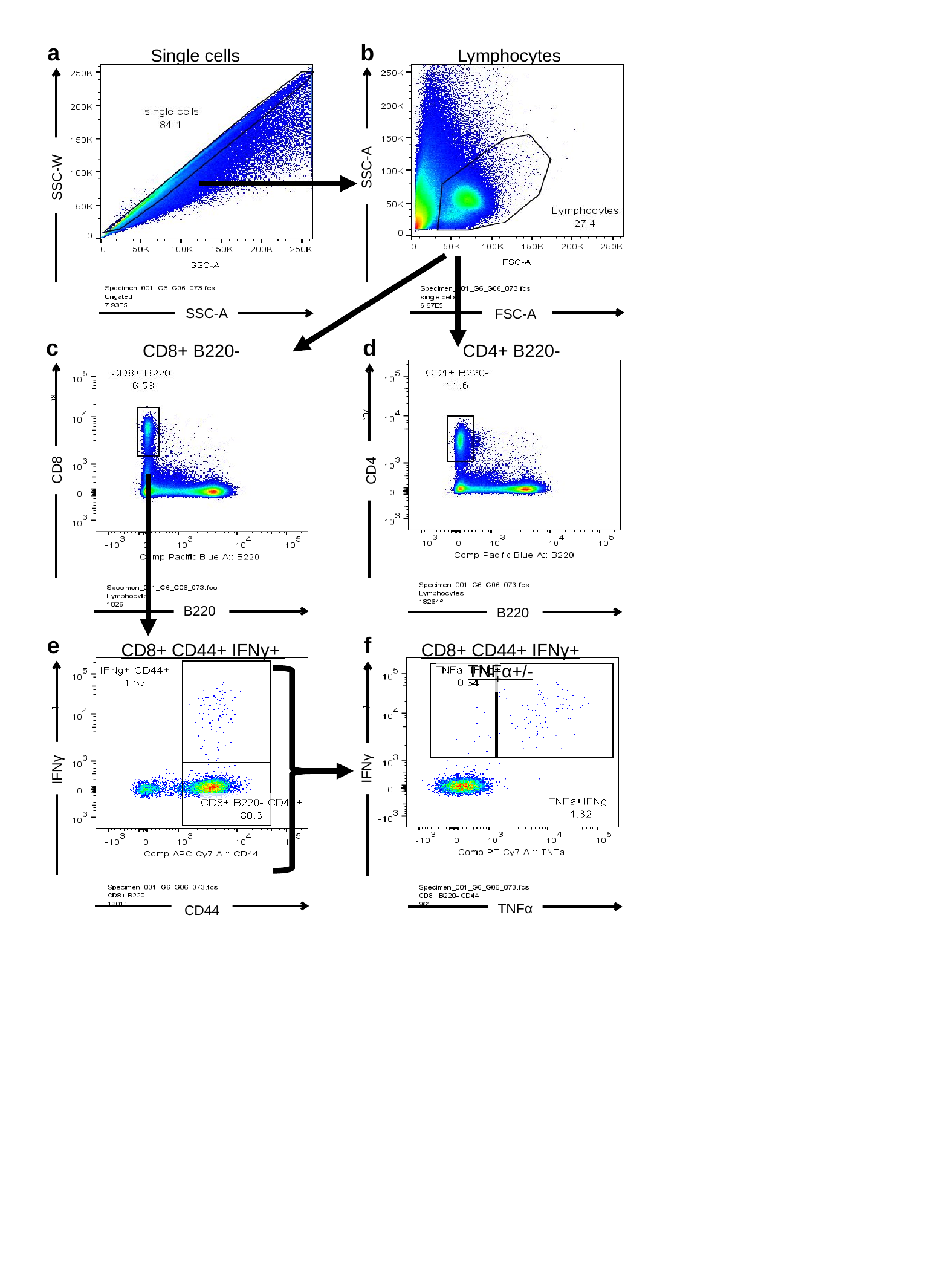

a
b
Single cells
Lymphocytes
SSC-A
SSC-W
SSC-A
FSC-A
c
d
CD8+ B220-
CD4+ B220-
CD8
CD4
B220
B220
e
f
CD8+ CD44+ IFNγ+
CD8+ CD44+ IFNγ+ TNFα+/-
+
IFNγ
IFNγ
TNFα
CD44
